# Impact of nurse-led home visits on medication management and use of medical screenings for adults with intellectual disabilities: a randomised-controlled trial

**DOI:** 10.1186/s12912-026-04317-4

**Published:** 2026-02-07

**Authors:** Christian Grebe, Stephan Nadolny, Sarah Palmdorf, Lisa Heitland, Meike Fechner, Annika Maaß, Änne-Dörte Latteck

**Affiliations:** 1 Institute for Educational and Health-Care Research in the Health Sector, Hochschule Bielefeld-University of Applied Sciences and Arts, Interaktion 1, 33619 Bielefeld, Germany; 2 Institute for History and Ethics of Medicine, Interdisciplinary Center for Health Sciences, Martin Luther University Halle-Wittenberg, Halle, Germany; 3 Nursing Science Staff Unit, Franziskus-Hospital-Haderberg, Georgsmarienhütte, Germany

**Keywords:** Intellectual disability, Learning disabilities, Medication management, Home visits, Counseling, Patient education, Clinical nurse specialists, Germany

## Abstract

**Background:**

People with intellectual disabilities suffer from several chronic diseases earlier and more often. At the same time, they use medical screenings to prevent such diseases very irregularly due to a variety of barriers (e.g., social, communicative). Additionally, they often have to manage complex medication regimens characterised by polypharmacy and psychotropic drugs. This complexity often leads to low medication adherence. This study aimed to improve medication adherence and the uptake of medical screenings in adult clients with intellectual disabilities.

**Methods:**

We conducted a randomised-controlled trial with waiting list and three months follow up in Germany. Clinical nurse specialists provided education to the clients and their caregivers about their medication and medical screenings during two home visits over a three-month period. They analysed the existing medication management and initiated changes where necessary. The control intervention was usual care. Inclusion criteria were: ICD diagnosis F70-79, ≥ 18 years, complex medication regimen (≥ 5 prescribed drugs) understanding of the German language. The primary outcome was medication adherence (MARS-D). Secondary outcomes were the use of medical screenings, complexity of the medication regimen, psychotropic medication, health-related quality of life and health status. Participants were randomised using block randomisation. Raters and analysts were blinded. Analysis was conducted via analysis of covariance (ANCOVA).

**Results:**

We recruited 162 participants, of whom 154 could be analysed. In the intervention group adherence changed from 24.7 ± 1.1 to 24.9 ± 0.4 and in control group from 24.8 ± 0.9 to 25.0 ± 1.2. A significant difference between the study groups (*p* > 0.05) was found only for the MRCI-D subscale “application forms” with a slightly lower complexity in the intervention group (η < 0.06, *p* < 0.05).

**Conclusions:**

Based on quantitative evidence nurse-led home visits did not improve medication adherence. In addition, there were no clinically meaningful changes in the secondary outcomes. The study aimed to recruit people with intellectual disabilities from a range of settings, but the vast majority of participants lived in the residential care setting. This resulted in people having high adherence at baseline. Furthermore, the three-month interval between the two visits may have been too short.

**Trial registration:**

German Clinical Trials Register DRKS00014101 (date registered: 17/08/2018).

## Background

People with intellectual disabilities have a higher risk of certain somatic diseases [1–3] and mental disorders than the general population [4, 5]. The risk for epilepsy is higher, with a prevalence of at least 20% in people with intellectual disabilities [1, 6], compared to 0.7% in the general population [7]. They also have higher rates of diabetes, overweight and obesity than the general population [1, 8].

Preventive health care is a viable option to address potential problems and prevent health issues from becoming chronic. However, people with intellectual disabilities have lower rates of receiving preventive health care. They are less likely than the general population to seek preventive health care, such as colorectal cancer or prostate cancer screening [9, 10]. Only a small proportion of women with intellectual disabilities attend gynecological screenings [11], and evidence on dental health and preventive care among people with intellectual disabilities also indicates inadequate preventive care practices.

Clients with intellectual disabilities often take medications throughout their lives to manage their underlying condition. Psychotropic medications are often part of the medication regimen, with one in two individuals taking at least one psychotropic medication [12, 13]. The number of medications increases with age and additional medical conditions (e.g., hypertension, diabetes mellitus). One-third [14] to three-quarters [15] of older people with intellectual disabilities have medication regimens characterised by polypharmacy. Polypharmacy is most commonly defined as the prescription of five or more drugs [16]. It increases the risk of prescription errors, side effects and drug interactions [17] as well as the overall risk of drug-related problems. Avoidable side effects, overdoses, and medications that are not or no longer needed are among the most common clinically relevant prescription errors among people with intellectual disabilities [15, 18, 19]. Mortality risk doubles with polypharmacy in people over 50 with an intellectual disability [14].

As the number of prescribed medications increases, medication-related adherence decreases [20, 21]. This is also true for people with intellectual disabilities [22, 23]. A factor influencing medication adherence may be the lower health literacy in people with intellectual disability [24]. It is sometimes unclear to them why they are taking certain medications and what they need to be aware of (e.g., symptoms of side effects or drug interactions).

Medication side effects are a well-recognised barrier to adherence, particularly among populations with communication or cognitive impairments. For individuals with intellectual disabilities, the ability to detect, articulate and report side effects may be limited, thus increasing the risk of nonadherence and adverse outcomes [25]. In addition, it is more difficult to communicate side effects due to different communication patterns. Because of these barriers in communication, side effects do not only influence willingness to continue medication but also complicate monitoring and shared decision-making [26].

Scheduled systematic reviews of medication regimens (medication reviews) can contribute to reduce the occurrence associated with medication-related problems. There have been positive experiences with the target group, especially with reviews that were conducted on a multi-professional basis [23, 27]. It is assumed that pharmacologically well-trained nurses are particularly well suited to identify drug-related problems because of their knowledge of diseases and symptoms [28]. Initial experience indicates that information and training tailored to people with intellectual disabilities can improve their understanding of their medication [29, 30] and, building on this, improve their medication-related adherence.

The goal of the intervention reported in this study was to optimise complex medication management and promote preventive health care for adults with intellectual disabilities. Clinical nurse specialists (CNS) should achieve this through outreach home visits. This is a rather new approach in Germany, as the use of CNS in the community setting is not common in Germany (see [31] for further information).

The study addressed the following primary research question: Can a complex intervention consisting of outreach, consultative home visits by CNS with a focused on the use of medication management and preventive health care improve adherence to prescribed medication among adults with intellectual disabilities compared to standard care, as well as the use of preventive medical screenings?

## Methods

### Trial design

To evaluate the effectiveness, we conducted a randomised controlled trial (RCT) in Germany with two measurement time points (t0 as baseline and t1 as follow-up after three months) and two groups (intervention group: IG and control group: CG) in a superiority design. The CG was established as a waiting list group. After completion of data collection at t1, the participants also received the intervention, but this was not part of the RCT. The study took place from February 2018 to May 2021. We report in this paper in accordance with the CONSORT 2010 statement.

### Intervention

**Fig. 1:**
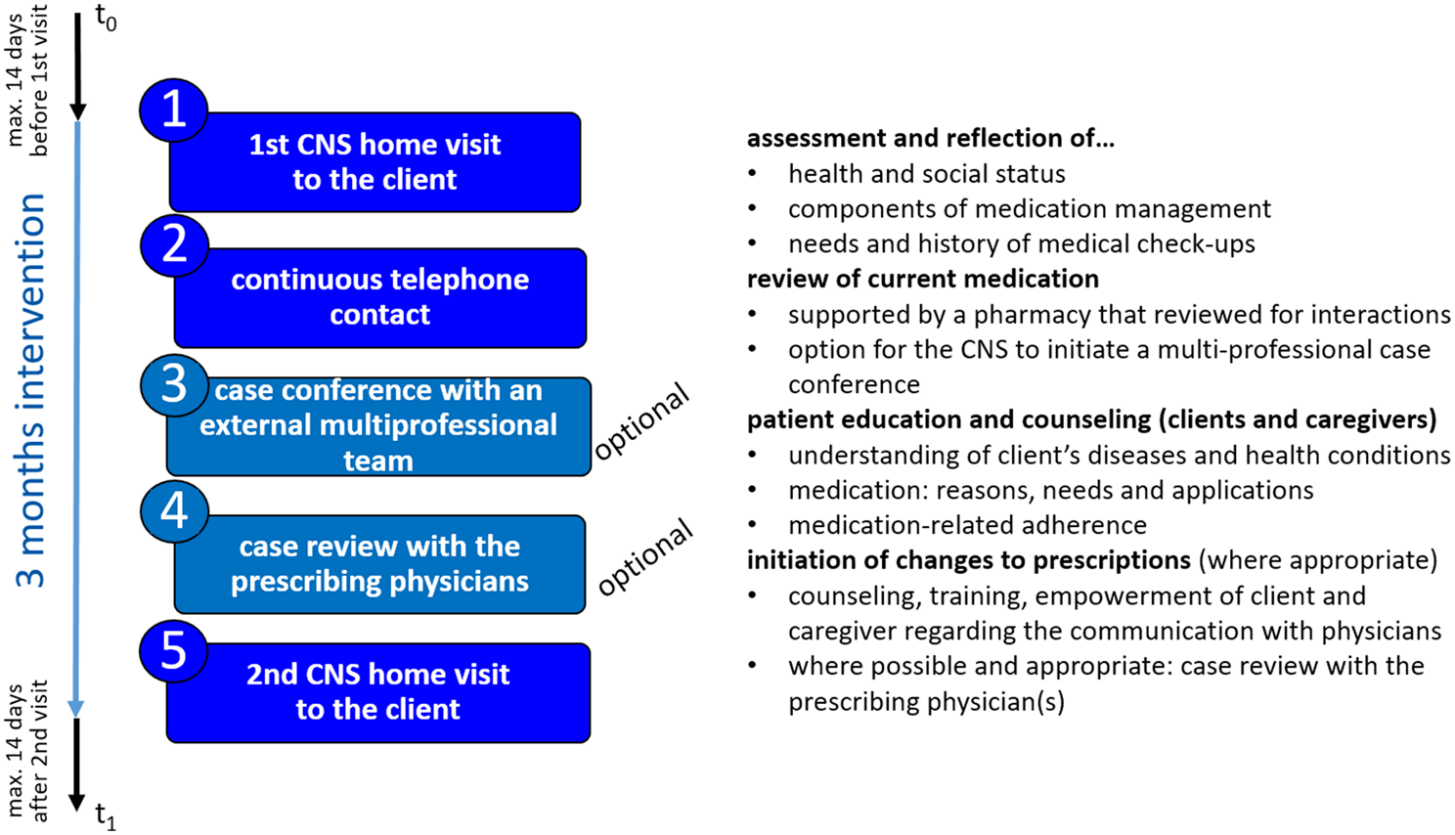
Steps of the intervention and measurement points

The intervention (Fig. 1) consisted of:

1. Two outreach home visits by a CNS (60–120 min each).
2. Continuous contact via telephone and/or e-mail between the CNS and the participant as well as with the formal and informal care system.
3. An optional interdisciplinary case conference.
4. An optional case review with the prescribing physicians.

The intervention was carried out over a three-month period. This timeframe was chosen based on feasibility constraints and because of funding timelines.

The CNSs received the participants’ medication plan for initial review prior to the first visit. During the first visit, the CNS thoroughly reviewed and analszed various components of the medication plan (e.g., appropriateness, side effects, correct intake times, interactions of medication) and medication management (e.g., procurement, safekeeping, intake, disposal) [23] with respect to the client’s health problems, investigated the extent to which health conditions could be possible side effects of the medication and discussed their findings with the clients. All participants were offered the option of having an additional medication review carried out by a pharmacy. Where this option was taken up, the findings were incorporated into the CNS analysis. Recommendations for adjustments to the medication plan were based on systematic evaluation criteria discussed during CNS training, including, for example, potential and investigated side effects, appropriateness of indication (alignment with clinical guidelines), dosage, and complexity. Recommendations were made in consultation with the client and their legal guardian.

The CNSs offered client education on medication management and medication adherence (e.g., on purpose of the medication, intake, storage) tailored to the individual’s preferences, communication patterns and context [30]. The content of the education varied widely among the clients, as some clients were very knowledgeable about the purpose of their medications but had problems taking them on time or storing them properly, while others were not aware of the purpose of each medication. Therefore, as is often the case with complex interventions, the intervention can only be standardised by function and not by form [32].

As part of the initial unstandardised assessment, the CNS also identified potential and prevalent health problems and identified appropriate medical screenings for which the client was eligible. Because polypharmacy and multimorbidity [33] as well as intellectual disabilities [34] are correlated, the CNS also educated the clients about prevention to (1) potentially avoid any exacerbation or chronification of diseases, (2) promote early detection of health problems, (3) increase understanding of prevention, and (4) reduce barriers to prevention. Topics covered included the purpose and importance of primary health care, access to services, the relationship between behavior and health and medical screenings [35–37]. Counseling was client-centered with predominantly passive involvement of the primary caregiver or staff members to promote the clients’ empowerment. It was obligatory that the changes were in accordance with the clients’ and, if applicable, their legal guardians, preferences.

In between the two visits, the CNS kept in touch with the clients as well as the formal and informal care system via telephone or e-mail. They provided further education if needed and answered questions regarding preventive health care and medication management. They contacted the prescribing physicians and proposed prescription changes when appropriate.

During the second visit, the CNS evaluated the changes made in the medication plan and medication management with the clients. The focus was on the clients’ wellbeing with regard to medication changes and their ability to manage their medications properly.

The CNSs used two internally developed schedules for the first and second home visit to ensure a consistent approach.

Another component of the intervention was the possibility to initiate an interdisciplinary case conference with an external team consisting of physicians, psychiatrists and pharmacists. For very complex medication regimens, this served as an open space to discuss uncertainties and find creative solutions for the particular case. Moreover, having an external perspective on the case besides the prescribing physicians might foster out-of-the box solutions. The CNS moderated the conference.

The final component of the intervention was an optional case review with the prescribing physicians. While there may be only brief contact between the CNS and physicians when suggesting medication changes in day-to-day interactions, in complex cases the CNS reviewed the entire medication plan with the prescribing physicians. This is especially important since the different medical disciplines often prescribe different medications and do not always know what the other disciplines have prescribed. This should serve as a longer case discussion to find the most appropriate solution or initiate communication between the different attending physicians.

Prior to the intervention, the CNS received 80 h of in-house training, developed specifically for this particular intervention. This consisted of theoretical input, discussion and role-playing on the following topics: CNS roles, communicative and behavioral challenges of clients with intellectual disabilities and strategies, easy language, home visits as intervention, counseling of people with intellectual disabilities, medical aspects of people with intellectual disabilities, International Classification of Functioning, Disability and Health, medical screenings, diagnostics and therapeutic interventions for people with intellectual disabilities from the perspective of the general practitioner, pharmacology, interactions of frequently prescribed pharmacological substances, psychotropics, medication plans, adherence, promotion of adherence, legal aspects [38].

### Participants

Inclusion criteria were a diagnosis of intellectual disability according to ICD-10 (F70-F79), age ≥ 18 years, understanding of the German language and the presence of a complex medication therapy defined as at least five medically prescribed medications [16] at the time of enrollment. Pro re nata (PRN) medications only included in any medication count of this study only if they had been taken in the previous three months. Exclusion criteria were uncertain legal guardianship, terminal illness or an acute infection requiring isolation.

Participants could live in any setting (residential care, group living, living alone), but our main focus was on recruiting people in the more independent settings, as we anticipated that there would be more problems with medication adherence than in the residential care setting.

### Recruitment, randomisation & blinding

We recruited participants by contacting service providers and institutions for care for people with intellectual disabilities in seven of Germany’s 16 federal states with a focus on North-Rhine-Westphalia and Hamburg. The other states in which we recruited were Schleswig-Holstein, Lower Saxony, Bremen, Mecklenburg-Western Pomerania and Berlin.

To inform about the intervention and the study, we held information events at our cooperation partners’ offices and institutions, which provide care and/or support for people with intellectual disabilities. In addition, we conducted structured telephone calls to similar service providers and institutions in order to identify additional potential participants and cooperation partners. Recruitment then took place in 2 ways: (1) Individuals and, if applicable, their legal guardians were approached directly and asked to participate, (2) The institution managers identified suitable potential participants and asked them and, if applicable, their legal representatives for permission to share their contact details with the study team. In both cases, a written and verbal explanation of the study ensued to obtain informed consent. The participants were randomised 1:1 to IG and CG using block randomisation with randomly permuted blocks of lengths 2, 4 and 6 [39] with the R-package “randomizeR” [40]. Participants received information about their group assignment by telephone from two coordinators responsible for scheduling data collection and intervention appointments.

Due to the characteristics of the intervention, it was not possible to blind the participants and CNS, but the raters who performed the outcome measurement were blinded. However, there was a risk that group membership could be mentioned or otherwise revealed by the participant or the participant’s caregiver during data collection. To measure the success of blinding, we asked the raters to estimate whether the case belonged to the IG or CG at the end of each data collection. The raters had to give one of the following reasons for the assessment: (1) Information from the participant, (2) Information from the caregiver, (3) Information from the legal guardian, (4) Information from documents (e.g., information about the intervention in the medication plan) and (5) no specific reason.

### Outcomes & data collection

The primary outcome was adherence related to the medication regimen, measured with the Medication Adherence Rating Scale (MARS-D) (0–25 points, higher points indicate better adherence) as a proxy assessment of the caregiver [41, 42].

Secondary outcomes were:

- adherence to the medication regimen, measured with the Medication Adherence Rating Scale (MARS), as self-assessment.
- health-related quality of life, measured by means of.
  - EQ-5D-3 L visual analog scale, as self-assessment. (0-100 points, higher points indicate higher quality of life)
  - EQ-5D-3 L score, as self-assessment.
  - EQ-5D-3 L visual analog scale, as proxy assessment of caregiver.
  - EQ-5D-3 L score, as a proxy assessment of the caregiver [43].
- medication regime complexity measured by the three sub scores (dosage forms, dosing frequency, additional dosing instructions) and the total score of the Medication Regime Complexity Index (MRCI-D) (0-infinite, higher points indicate higher complexity) [44, 45].
- number of psychotropic medication. We considered the following fourth-level chemical/therapeutic/pharmacological subgroups of the Anatomical Therapeutic Chemical Classification System 7 (ATC) as psychotropics: Anticholinergic agents for Parkinson (N04A), Dopaminergic agents (N04B), Antipsychotic drugs (N05A), Anxiolytic drugs (N05B), Hypnotics and sedatives drugs (N05C), Antidepressant drugs (N06A), Psychostimulants, agents used for ADHD and nootropics (N06B), Psycholeptics and Psychoanaleptics in combination (N06C), Dementia drugs (N06D) and Antiepileptic drugs (N03A).
- health status measured by 36-item WHODAS 2.0 (proxy version; 0-100 points, with 100 indicating complete disability) [46].
- utilisation of at least one preventive medical screening in the last three months. This was the only self-developed item for outcome measurement (“Have you had a check-up in the last 3 months?”; response format: yes/no). The following screenings were considered:
  - abdominal aortic aneurysm.
  - breast cancer.
  - cervical cancer.
  - chlamydia.
  - colorectal cancer.
  - dental.
  - general health check-up.
  - prostate cancer.
  - skin cancer.

The respective German version was used for all of the above mentioned assessment instruments. All data was measured at baseline (t0), with a maximum of 14 days prior to the first visit of the CNS and at follow-up after three months (t1), also with a maximum of 14 days after the end of the intervention.

The questionnaire was pre-tested in a group of people with intellectual disabilities. The pretest helped us to determine whether respondents understood the questions. The raters received training in communication skills (easy read and plain language), interviewing people with intellectual disabilities and had prior experience in health or social care.

We collected data through structured face-to-face interviews with the participants and their primary caregiver and by reviewing medication records. With the onset of the COVID-19 pandemic in March 2020 the face-to-face interviews were conducted via video conferencing software hosted on the servers of Bielefeld University of Applied Sciences and Arts or alternatively by telephone.

### Ethical aspects

All methods in this study were performed in accordance with the Declaration of Helsinki. This study was reviewed and approved by the ethics committee of the Competence Center Gesundheit (CCG, approval number 2018-11). Written and verbal informed consent has been obtained from the client and additionally from the legal guardian, if applicable.

### Adverse events

Deaths, emergency hospitalizations and life-threatening situations were considered potential serious adverse events (SAEs). The protocol required that SAEs had to be documented reported to and reviewed by the principal investigator for a direct or indirect relationship to the intervention. The protocol required the principal investigator to decide whether to (dis-) continue the study after each SAE and to document the reasons for the decision.

### Statistical analysis

Outcomes were analysed using ANCOVAs with the respective follow-up measure as dependent variable, baseline measure as covariate and group assignment as fixed factor. Utilisation of medical screenings was only measured at follow-up and analysed using an independent samples t-test. We compared baseline characteristics using independent samples two-sided t-tests, Fisher’s exact tests and chi^2^-tests, depending on the scale of measurement. Data analysis followed the intention-to-treat principle. IBM SPSS 25 was used for the statistical analysis.

The significance levels of outcomes that were tested under the same global hypothesis were adjusted using the Bonferroni-method. The adjusted local significance levels for each individual test were α = 0.025 for the two outcomes measured with the MARS-D and α = 0.0125 for the four outcomes measured with the EQ-5D as well as for the four outcomes measured with MRCI-D.

G*Power software [47] was used for a priori power calculation. We calculated with a Bonferroni-adjusted alpha of 0.025 for the primary outcome, a power of 0.8 and an effect size of f = 0.25. According to this analysis, *N* = 156 participants were required at follow up. We calculated a pessimistic drop-out rate of 30%, therefore *N* = 202 participants were targeted for recruitment.

## Results

### Sample characteristics

**Fig. 2:**
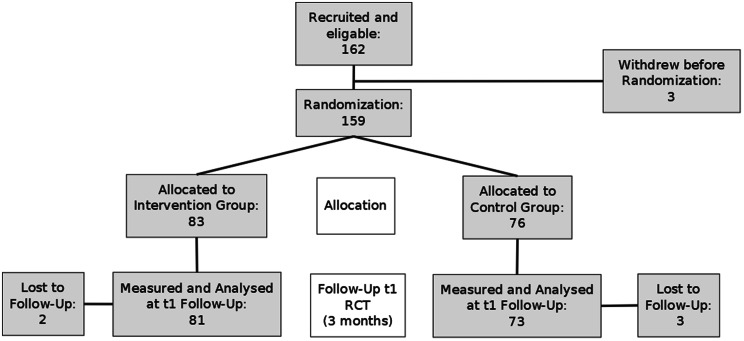
CONSORT flow chart

We recruited *N* = 162 participants into the study. Because three of them withdrew their consent before randomisation, *N* = 159 participants were randomised and allocated to the two study groups. The drop-out rate was 3.1% and therefore *N* = 154 participants were analysed at t1 (Fig. 2).

The mean age of the participants was 53.2 years (standard deviation (SD): 12.3), ranging from 22 to 85 years. The proportion of women and men was exactly the same (50.0%), but there were slightly (but not significantly) more women in the intervention group (Table 1). The majority lived in residential care facilities (87.5%), with smaller proportions living in group homes (9.9%) or alone (2.6%). All of the participants received daily support and 21.7% received informal support from family members or other caregivers (complementary to that provided by professionals).

**Table 1:** Baseline characteristics

characteristic		IG (*N* = 83)	CG (*N* = 73)	*p*-value
age (mean ± SD)		54.3 ± 14.1	52.6 ± 12.4	0.422^a^
sex (female) n (%)		45 (54.2)	33 (43.4)	0.115^b^
living situation	alone group Home residential care	5 (6.1) 7 (8.5) 79 (85.4)	0 (0.0) 9 (12.0) 66 (89.0)	0.079^c^
support through family, friends or neighbors		21 (25.6)	13 (17.3)	0.247^b^
number of medications at t0 (mean ± SD)		8.5 ± 3.3	8.2 ± 3.1	0.558^a^

SD = Standard deviation, IG = intervention group, CG = control group

^a^ Independent samples t-test, ^b^ Fisher’s exact test, ^c^ Chi^2^ test

Approximately half of the sample had moderate to extreme problems with concentrating, remembering to do important things, understanding and in starting and maintaining a conversation. A third of the sample had no problems with these four tasks. About 40% had at least moderate problems with finding solutions in everyday life and learning new tasks, but only 13.5% of the sample had no problems with these tasks. With regard to these six items of the WHO-DAS 2.0, there were no statistically significant differences between the study groups (Table 2).

**Table 2:** Baseline responses to WHO-DAS 2.0 cognition items, median category highlighted

	group	N ^a^	none	mild	moderate	severe	extreme or cannot do	*p*-value^b^
			relative frequencies					
concentrating on doing something for ten minutes	IG	83	34.9%	**16.9%**	22.9%	14.5%	10.8%	0.388
	CG	76	26.3%	19.7%	**27.6%**	13.2%	13.2%	
remembering to do important things	IG	79	32.9%	**17.7%**	20.3%	8.9%	20.3%	0.478
	CG	76	36.8%	**15.8%**	21.1%	15.8%	10.5%	
analysing and finding solutions to problems in day-to-day life	IG	81	16.0%	19.8%	**25.9%**	13.6%	24.7%	0.303
	CG	75	10.7%	13.3%	**30.7%**	22.7%	22.7%	
learning a new task, for example, learning how to get to a new place	IG	80	15.0%	16.3%	**33.8%**	15.0%	20.0%	0.253
	CG	75	18.7%	22.7%	**29.3%**	13.3%	16.0%	
generally understanding what people say	IG	83	38.6%	**22.9%**	25.3%	9.6%	3.6%	0.890
	CG	76	35.5%	**27.6%**	23.7%	7.9%	5.3%	
starting and maintaining a conversation	IG	83	36.1%	**18.1%**	15.7%	12.0%	18.1%	0.821
	CG	76	38.2%	**15.8%**	17.1%	13.2%	15.8%	

IG = intervention group, CG = control group, ^a^ *n* < 83 for IG and < 76 for CG indicate missing values (participants did not answer the respective question), ^b^ Mann-Whitney-U-test, bold values indicate median category

The mean number of prescribed and non-prescribed medications at baseline was 8.4 (SD: 3.2), with a maximum of 22 medications. Overall participants received various psychotropics. The most prevalent psychotropics were antipsychotic, antiepileptic and antidepressant drugs (see Table 3 for further information on the distribution of psychotropics in the IG and CG).

**Table 3:** Prescribed psychotropics at baseline

psychotropics	frequency		*p*-value ^a^
	IG	CG	
anticholinergic agents for Parkinson	4 (4.8%)	7 (9.2%)	0.354
dopaminergic agents	4 (4.8%)	1 (1.3%)	0.369
antipsychotic drugs	45 (54.2%)	47 (61.8%)	0.340
anxiolytic drugs	14 (16.9%)	14 (18.4%)	0.837
hypnotics and sedatives drugs	4 (4.8%)	6 (7.9%)	0.521
Antidepressant drugs	30 (36.1%)	26 (34.2%)	0.869
psychostimulants, agents used for ADHD and nootropics	1 (1.2%)	0 (0.0%)	1.000
psycholeptics and psychoanaleptics in combination	0 (0.0%)	0 (0.0%)	n.a.
dementia drugs	1 (1.2%)	3 (3.9%)	0.349
antiepileptic drugs	41 (49.4%)	35 (46.1%)	0.751

IG = intervention group, CG = control group; ^a^ Fisher’s exact test

### Utilisation of the intervention

Optional interdisciplinary case conferences were convened for six clients of the IG. For seven IG clients, the CNS discussed medications in person with the prescribing physician. Due to contact restrictions related to the COVID-19 pandemic, we replaced home visits with video calls for seven participants.

Which one of the three CNSs provided the intervention had no significant effect on any of the measured outcomes in the intervention group (Table 4).

**Table 4:** Impact of the concrete clinical nurse specialist (CNS) providing the intervention on the outcomes (Intervention group only)

outcome	*N*	CNS-effect (*p*-value)
MARS-D (proxy-rating)	80	0.901
MARS-D (self-rating)	55	0.429
EQ-5D-3 L index (self-rating)	53	0.921
EQ-5D-3 L index (proxy-rating)	80	0.878
EQ-5D-VAS (self-rating)	31	0.789
EQ-5D-VAS (proxy-rating)	79	0.854
WHODAS-t32 (proxy-rating)	72	0.636
MRCI-D dosage forms	81	0.143
MRCI-D dosing frequency	81	0.430
MRCI-D additional dosing instructions	81	0.738
MRCI-D total score	81	0.618
number of prescribed psychotropics	81	0.957
preventive medical screening	80	0.999

### Blinding of the raters

At baseline, the raters’ assessment of whether a participant belonged to the IG or CG was incorrect for 38.6% of participants and at follow-up for 44.7% of the participants. In 83.3% of the cases the raters stated that there was no specific reason for their estimation (just guessing), in 11.9% the group membership was revealed by the caregivers and in 3,9% by the participants themselves. In 1.0% of the cases, there were other reasons for the raters that led to their estimation of group membership. We consider these values as a successful blinding of the outcome measurement.

### Medication adherence

Adherence was already high on baseline with a mean of 24.7 points in the IG and 24.8 points in the CG as measured with the MARS-D, which has a maximum score of 25. There was no statistically significant group effect (Table 5).

### Health-related quality of life

In self-rating, the scores of EQ-5D’s visual analogue scale decreased by -6.7 points in the IG and by -9.1 points in the CG. The EQ-5D-3L-index decreased by -6,8 points in the IG and by -0.7 points in the CG.

In the proxy rating, the index score changed by -1.1 points in the IG and by + 1.2 points in the CG. The visual analogue score changed by 2.4 points in the IG and by + 0.01 points in the CG. The group effects were not significant (Table 5).

### Health and disability status

From baseline to follow-up the WHODAS-32-score changed by -0.3 points in the IG and by -2.6 points in the CG. There was no significant group effect (*p* = 0.695) (Table 5).

### Complexity of the medication regimen

The MRCI-D total score decreased by 0.55 points in the IG and by 0.07 points in the CG (*p* = 0.450). The MRCI-D dosage form sub score decreased by 0.44 points in the IG and increased by 0.35 points in the CG. The group effect (*p* = 0.019) was significant at the Bonferroni-adjusted alpha-level with an effect size of η^2^ = 0.036. The dosing frequency sub score decreased by 0.40 points in the IG while it increased by 0.42 points in the CG. The sub score for additional dosing instructions increased by 0.16 points in the IG and decreased by 0.67 points in the CG. None of the group effects was significant (Table 5).

### Prescribed psychotropics

At baseline, 86.4% of the participants in the IG and 87.7% in the CG had at least one prescribed psychotropic drug in their medication regimen. The most often prescribed substance groups were antipsychotics, antiepileptics and antidepressants (Table 3).

The mean number of prescribed psychotropics did not change between baseline and follow-up, in either the IG or the CG (Table 5). The group effect was not significant.

### Medical screenings

At follow-up, 57.5% of the participants in the CG and 68.1% of the IG had a non-acute medical screening in the past three months, according to their caregivers, so the relative risk for not participating in a medical screening was 1.33, but this effect was not statistically significant (*p* = 0.240).

No serious adverse events occurred during the study.

**Table 5:** Primary and secondary outcomes

outcome	group	N	mean ± SD (95% CI)		group effect (p-value)
			T0	T1	
MARS-D (proxy-rating) (0–25 pts., higher scores indicate better adherence)	IG	80	24.7 ± 1.1 (24.4–24.9)	24.9 ± 0.4 (24.8–25.0)	0.639
	CG	72	24.8 ± 0.9 (24.7–25.0)	25.0 ± 1.2 (24.9–25.0)	
MARS-D (self-rating)	IG	55	24.5 ± 1.4 (24.0-24.9)	24.9 ± 0.4 (24.7–25.0)	0.347
	CG	55	24.5 ± 1.2 (24.2–24.9)	24.9 ± 0.3 (24.9–25.0)	
EQ-5D-3 L index (self-rating) (0-100 pts., higher scores indicate better quality of life)	IG	53	71.7 ± 22.0 (66.7–79.1)	64.9 ± 25.4 (57.2-71-2)	0.136
	CG	56	69.5 ± 25.3 (62.3–76.9)	68.8 ± 25.2 (62.2–75.7)	
EQ-5D-3 L index (proxy-rating)	IG	80	59.8 ± 25.2 (53.9–65.2)	57.7 ± 25.4 (52.0-63.4)	0.208
	CG	72	57.6 ± 25.0 (51.3–62.3)	59.8 ± 24.2 (54.1–65.5)	
EQ-5D-VAS (self-rating) (0-100 pts., higher scores indicate better quality of life)	IG	31	72.6 ± 20.9 (63.3–78.0)	65.9 ± 25.3 (57.1–75.9)	0.242
	CG	30	70.8 ± 23.0/ (63.1–80.0)	61.7 ± 21.5 (51.3–68.0)	
EQ-5D-VAS (proxy-rating)	IG	79	70.9 ± 21.2 (65.7–75.3)	68.5 ± 20.4 (63.7–72.8)	0.894
	CG	71	67.5 ± 19.6 (63.1–72.4)	67.6 ± 18.1 (63.6–72.2)	
WHODAS-32 (proxy-rating) (0-100 pts., higher scores indicate higher disability)	IG	72	45.0 ± 23.4 (40.2–51.6)	44.7 ± 23.7 (38.5–49.8)	0.695
	CG	65	46.4 ± 22.5 (40.2–51.5)	42.8 ± 20.7 (38.2–48.5)	
MRCI-D dosage forms (0–85 pts., higher scores, indicate greater complexity)	IG	81	3.9 ± 3.0 (3.2–4.5)	3.4 ± 2.9 (2.8-4.0)	0.019
	CG	73	3.9 ± 3.2 (3.2–4.7)	4.3 ± 3.4 (3.5–5.1)	
MRCI-D dosing frequency (0-infinite pts., higher scores indicate greater complexity)	IG	81	12.1 ± 5.9 (10.8–13.4)	11.7 ± 5.5 (10.5–13.0)	0.054
	CG	73	12.1 ± 5.3 (10.9–13.4)	12.6 ± 6.0 (11.2–14.0)	
MRCI-D additional dosing instructions (0-infinite pts., higher scores indicate greater complexity)	IG	81	5.9 ± 5.2 (5.7–7.1)	6.1 ± 5.7 (4.8–7.3)	0.690
	CG	73	7.1 ± 6.8 (5.5–8.7)	6.5 ± 5.1 (5.3–7.7)	
MRCI-D total score (0-infinite pts., higher scores indicate greater complexity)	IG	81	21.7 ± 11.2/ (19.3-24-2)	21.2 ± 11.0 (18.8–23.6)	0.453
	CG	73	23.2 ± 12.5 (20.8–26.1)	23.1 ± 12.0 (20.3–25.9)	
number of prescribed psychotropics	IG	81	2.6 ± 1.7 (2.2-3.0)	2.6 ± 1.8 (2.2-3.0)	0.631
	CG	73	2.7 ± 2.0 (2.3–3.2)	2.7 ± 2.0 (2.3–3.2)	

SD = standard deviation, 95% CI = 95% confidence interval, IG = intervention group, CG = control group

## Discussion

We conducted an RCT to improve medication adherence, quality of life, medication complexity as well as the uptake of prevention programs in people with intellectual disabilities through a complex intervention involving outreach home visits by CNS. We were unable to identify a statistically or clinically meaningful effect of the intervention for the majority of our chosen outcomes. Interestingly, the dropout rate was considerably lower than anticipated. This may reflect high commitment among institutions or caregivers once initial consent was provided.

Despite the lack of significant effects, our findings provide valuable insight into the contextual and structural barriers to intervention effectiveness in institutionalised populations with intellectual disabilities. This adds to the understanding of how setting-specific characteristics affect intervention success. Regarding the lack of effect of the intervention on the medication adherence, one explanation for this was the already extremely high level of adherence of the participants at baseline. From a statistical perspective, we would have needed a larger sample size to observe an effect of this smaller size than anticipated. From a practice perspective, we wanted to include people with intellectual disabilities regardless of the setting (living in residential care homes, living groups or on their own) but with a strong focus on more independently living people; most of the participants were living in residential care homes. Similar to people in other long-term care settings, these residents often receive their medication from their caregivers. They might check their medication with regards to the correctness and indicate omissions or changes (e.g., the blue pill is missing) [48, 49], but they comply with taking their medication. This behavior might be similar for people with intellectual disabilities and even more prevalent, as people with intellectual disabilities who live in residential institutions in Germany do so for a longer period of time [50–52]. The reported behavior was also a topic that was frequently raised by caregivers in the institutions. It may be related to a high level of trust between caregivers and their clients as well as to a delegation of the competence on medication to their caregivers. However, the adequate taking of the medication is basically not adherence, but rather compliance [53].

Although we used a validated assessment of medication adherence in the MARS-D, it focuses more on medication taking behavior [54] and therefore we may have actually measured compliance in this sample. Additionally, although MARS-D is validated in general populations, its validity for people with ID is uncertain. It was used for outcome measurement in the absence of more appropriate tools. We did not additionally measure health literacy with regard to medication in this population due to the lack of a reliable German instrument. For future studies, suitable medication adherence assessments should be developed using participatory research methods specifically for the population of people with intellectual disabilities.

A key limitation of our study was the mismatch between the target population and the primary outcome measure. Most participants resided in institutional settings where medication adherence is largely enforced through care protocols. This led to ceiling effects and limited the room for intervention-driven improvements. Furthermore, studies on medication adherence among people with intellectual disabilities have shown lower adherence rates although using different measures such as the Medication Possession Ratio for about 80% of the participants having good adherence for diabetic medication [55] and about 50% for antihypertensive medication [56, 57]. Had we used these measures, our adherence rates would have been even higher, since most people lived in the residential care setting with a centralised medication distribution.

The mean number of medications with 8.4 is lower than in two smaller studies on people with intellectual disabilities in a Swiss hospital (9.4) [34], in the Dutch residential care setting (Median = 10) [58] and in a well-sized study from a US internal medicine clinic (11.2) [59]. It is higher than in well-sized longitudinal Irish studies on older people with intellectual disabilities IDS-TILDA in wave 1 (5.7) [60] and wave 2 (Median = 7) [61] as well as an Australian longitudinal study (Median = 3) [62].

The higher numbers in the smaller studies could be due to a selective sample, since both studies focused on polypharmacy although Lonchampt et al. [34] did not define it as an inclusion criterion. The lower numbers in the other studies can be explained by the lack of polypharmacy as a criterion. Overall, the number of medications is between those of the smaller studies with polypharmacy and those without.

The proportion of psychotropic medication in our sample is extremely high with 86.4% of the participants in the IG and 87.7% in the CG. This is higher than in comparable studies, which range from 32.2% [63] to 67.5% [59]. An explanatory factor for these high values is that our study focused on polypharmacy and many participants lived in residential care settings.

The mean MRCI-D score is comparable to the IDS TILDA (22.5) [64], lower than in the aforementioned US study (29.0) [59] and higher than in a smaller study on patients with autism spectrum disorder and intellectual disability (20.0) [65]. We did not observe a desired change in the complexity other than the dosage forms.

An effect on the prescribed drug regimens was made more difficult on the one hand by the fact that the CNS were involved in the discussions with the prescribed physicians in only a few cases. In most cases, the participants made the physician’s appointments themselves and, if necessary, attended them with their caregivers. This, of course, is generally desirable. However, this requires that people with intellectual disabilities and, where appropriate, their caregivers have a sufficient level of medication-related health literacy to be able to understand complex medical and pharmacological issues.

Some physicians also refused to collaborate with the CNS, and others, especially the neurologists, mentioned that the medication regime of psychotropic drugs took a long time to be calibrated and therefore objected to intervene. Some medication changes even required the people with intellectual disability to be hospitalised. Consequently, those changes would have only become apparent after the study was completed. Additionally, medication regimes were often developed by different physicians, because each physician prescribed medications for his field of expertise. It was difficult for the CNS to change or intervene in those structures, but this interdisciplinary communication is essential for a successful intervention [23]. A possible explanation for this might be the lack of experience in changing structures. We wanted to employ nurses with a master’s degree, however, this was not possible due to the German educational system for nurses, which is still strongly focused on graduating from vocational schools with fewer opportunities to study nursing as a primary qualification on an academic level. Many nurses, who wish to qualify academically complete three years at a vocational nursing school and then enroll in bachelor’s and subsequently master’s programs. As a result, there are only few graduates on a master’s level, who have the opportunity to work in a clinical setting. Most are employed in management, science or education. This is slowly but surely changing nowadays.

Our CNS had bachelor’s degrees and expertise in caring for people with intellectual disabilities, but we believe that a master’s level expertise gives more competence in analysing structural problems as well as overcoming those barriers. Therefore, changing structural problems in individual cases, which should rather be seen as a distant goal, might have been too demanding for the nursing experts.

We were not able to achieve improvements in quality of life or functional ability. This might be directly related to (a) the rather short follow-up period of three month - it is likely that a longer intervention and observation period would have been required to detect sustainable behavioral or clinical changes, especially regarding medication complexity and adherence - and (b) the minor changes in medication observed. One explanation for the slight decrease in health-related quality of life could be the lockdowns during the COVID-19 pandemic, which led to the temporary closure of sheltered workshops, other workplaces as well as social structures.

The exact content and frequency of educational contacts were not systematically recorded. Future studies should implement structured monitoring tools to assess intervention fidelity.

The CNS were not part of the organizational structures of the organizations caring for the participants. They came to the study participants as external consultants and experts. On the one hand, this approach has advantages due to the independence and the perspective unencumbered by internal structures. On the other hand, in this role a deeper understanding of the individual cases can only be achieved to a limited extent, and initiated change processes cannot be accompanied, or only over a short period. Therefore, it seems to be necessary to integrate the CNS’ nursing expertise at the provider level in the future.

In summary, we cannot report on a clinically relevant intervention from a quantitative perspective. We will publish the results of the qualitative study, which shows a different perspective in another paper.

### Limitations

The calculated sample size at baseline was *n* = 202, which was not achieved. We only recruited *N* = 162 and were able to analyse *n* = 154 participants. As the number of drop-outs (3.1%) was lower than expected (30%), the low recruitment number did not have too much of an impact. However, we missed the required sample size for analysis by two people. Mainly due to the impact of the COVID-19 pandemic, which made it difficult to recruit new participants. The sample is also not representative of the target population because of the oversampling of people living in residential care settings. However, it is quite difficult to draw a representative sample in the population anyway, as intellectual disabilities vary considerably in terms of people’s competencies and difficulties in everyday life.

Additionally, we had missing of data especially in the self-reported secondary outcomes of health-related quality of life, as well as medication adherence. However, we do not believe that this has changed the outcome of the study due to the lack of the effect or its magnitude.

## Conclusions

A complex intervention consisting of two home visits carried out by CNS including client education and medication review plus further optional interdisciplinary case discussions over three months resulted in no clinically meaningful effects on medication adherence, health-related quality of life, health, the number of psychotropic medication and utilisation of medical screenings. The intervention was one of the first studies on CNS for people with intellectual disabilities in Germany; however, the three-month period may have been too short. It was particularly difficult to recruit participants from the community setting for this study. Further studies in this setting should plan a longer period for recruiting participants.

## Data Availability

The datasets generated and analyzed during the current study are not publicly available due to prior commitments to not openly share the data, but are available from the corresponding author on reasonable request.

## Abbreviations

ANCOVA: Analysis of covariance
CG: Control group
CNS: Clinical nurse specialist
EQ5D: Euroquol 5D
IG: Intervention group
MARS-D: Medication Adherence Rating Scale – German Version
MRCI-D: Medication Regime Complexity Index – German Version
n.a.: not applicable
RCT: Randomised-controlled trial
VAS: Visual analogue scale
WHODAS: WHO Disability Assessment Schedule

## References

[1] Liao P, Vajdic C, Trollor J, Reppermund S. Prevalence and incidence of physical health conditions in people with intellectual disability - a systematic review. PLoS ONE. 2021;16:e0256294. 10.1371/journal.pone.0256294. 34428249 10.1371/journal.pone.0256294 PMC8384165

[2] van den Bemd M, Cuypers M, Bischoff EWMA, Heutmekers M, Schalk B, Leusink GL. Exploring chronic disease prevalence in people with intellectual disabilities in primary care settings: A scoping review. J Appl Res Intellect Disabil. 2022;35:382–98. 10.1111/jar.12957. 34750946 10.1111/jar.12957 PMC9298833

[3] Iwanaga K, Wu JR, Chan F, Rumrill P, Wehman P, Brooke VA, et al. A systematic review of systematic reviews of secondary health conditions, health promotion, and employment of people with intellectual disabilities. Aust J Rehabil Couns. 2021;27:13–40. 10.1017/jrc.2021.2.

[4] Buckles J, Luckasson R, Keefe E. A systematic review of the prevalence of psychiatric disorders in adults with intellectual Disability, 2003–2010. J Ment Health Res Intellect Disabil. 2013;6:181–207. 10.1080/19315864.2011.651682.

[5] Mazza MG, Rossetti A, Crespi G, Clerici M. Prevalence of co-occurring psychiatric disorders in adults and adolescents with intellectual disability: A systematic review and meta-analysis. J Appl Res Intellect Disabil. 2020;33:126–38. 10.1111/jar.12654. 31430018 10.1111/jar.12654

[6] Robertson J, Hatton C, Emerson E, Baines S. Prevalence of epilepsy among people with intellectual disabilities: A systematic review. Seizure. 2015;29:46–62. 10.1016/j.seizure.2015.03.016. 26076844 10.1016/j.seizure.2015.03.016

[7] Fiest KM, Sauro KM, Wiebe S, Patten SB, Kwon C-S, Dykeman J, et al. Prevalence and incidence of epilepsy: A systematic review and meta-analysis of international studies. Neurology. 2017;88:296–303. 10.1212/WNL.0000000000003509. 27986877 10.1212/WNL.0000000000003509 PMC5272794

[8] Ranjan S, Nasser JA, Fisher K. Prevalence and potential factors associated with overweight and obesity status in adults with intellectual developmental disorders. J Appl Res Intellect Disabil. 2018;31(Suppl 1):29–38. 10.1111/jar.12370. 28544175 10.1111/jar.12370

[9] Chauhan U, Kontopantelis E, Campbell S, Jarrett H, Lester H. Health checks in primary care for adults with intellectual disabilities: how extensive should they be? J Intellect Disabil Res. 2010;54:479–86. 10.1111/j.1365-2788.2010.01263.x. 20576060 10.1111/j.1365-2788.2010.01263.x

[10] Osborn DPJ, Horsfall L, Hassiotis A, Petersen I, Walters K, Nazareth I. Access to cancer screening in people with learning disabilities in the UK: cohort study in the health improvement network, a primary care research database. PLoS ONE. 2012;7:e43841. 10.1371/journal.pone.0043841. 22952783 10.1371/journal.pone.0043841 PMC3430609

[11] Lewis MA, Lewis CE, Leake B, King BH, Lindemann R. The quality of health care for adults with developmental disabilities. Public Health Rep. 2002;117:174–84. 10.1016/S0033-3549(04)50124-3. 12357002 10.1016/S0033-3549(04)50124-3 PMC1497422

[12] Chitty KM, Evans E, Torr JJ, Iacono T, Brodaty H, Sachdev P, Trollor JN. Central nervous system medication use in older adults with intellectual disability: results from the successful ageing in intellectual disability study. Aust N Z J Psychiatry. 2016;50:352–62. 10.1177/0004867415587951. 26019276 10.1177/0004867415587951

[13] Sheehan R, Hassiotis A, Walters K, Osborn D, Strydom A, Horsfall L. Mental illness, challenging behaviour, and psychotropic drug prescribing in people with intellectual disability: UK population based cohort study. BMJ. 2015;h4326. 10.1136/bmj.h4326. 10.1136/bmj.h4326 PMC4556752 26330451

[14] Schoufour JD, Oppewal A, van der Maarl HJK, Hermans H, Evenhuis HM, Hilgenkamp TIM, Festen DA. Multimorbidity and polypharmacy are independently associated with mortality in older people with intellectual disabilities: A 5-Year Follow-Up from the HA-ID study. Am J Intellect Dev Disabil. 2018;123:72–82. 10.1352/1944-7558-123.1.72. 29281324 10.1352/1944-7558-123.1.72

[15] Zaal RJ, van der Kaaij ADM, Evenhuis HM, van den Bemt PMLA. Prescription errors in older individuals with an intellectual disability: prevalence and risk factors in the healthy ageing and intellectual disability study. Res Dev Disabil. 2013;34:1656–62. 10.1016/j.ridd.2013.02.005. 23501585 10.1016/j.ridd.2013.02.005

[16] Masnoon N, Shakib S, Kalisch-Ellett L, Caughey GE. What is polypharmacy? A systematic review of definitions. BMC Geriatr. 2017;17:230. 10.1186/s12877-017-0621-2. 29017448 10.1186/s12877-017-0621-2 PMC5635569

[17] Stortz JN, Lake JK, Cobigo V, Ouellette-Kuntz HMJ, Lunsky Y. Lessons learned from our elders: how to study polypharmacy in populations with intellectual and developmental disabilities. Intellect Dev Disabil. 2014;52:60–77. 10.1352/1934-9556-52.1.60. 24635692 10.1352/1934-9556-52.1.60

[18] Thomson A, Roberts P, Bittles A. Navigating the maze: ethics approval pathways for intellectual disability research. J Med Ethics. 2014;40:782–6. 10.1136/medethics-2012-100899. 23963255 10.1136/medethics-2012-100899

[19] O’Dwyer M, McCallion P, McCarron M, Henman M. Medication use and potentially inappropriate prescribing in older adults with intellectual disabilities: a neglected area of research. Ther Adv Drug Saf. 2018;9:535–57. 10.1177/2042098618782785. 30181861 10.1177/2042098618782785 PMC6116771

[20] Lyles A, Culver N, Ivester J, Potter T. Effects of health literacy and polypharmacy on medication adherence. Consult Pharm. 2013;28:793–9. 10.4140/TCP.n.2013.793. 24322963 10.4140/TCP.n.2013.793

[21] Schwartz JK, Unni E. Inclusion of people with disabilities in research to improve medication adherence: A systematic review. Patient Prefer Adherence. 2021;15:1671–7. 10.2147/PPA.S314135. 34345167 10.2147/PPA.S314135 PMC8324980

[22] Tan X, Marshall VD, Balkrishnan R, Patel I, Chang J, Erickson SR. Psychotropic medication adherence among Community-Based individuals with developmental disabilities and mental illness. J Ment Health Res Intellect Disabil. 2015;8:1–22. 10.1080/19315864.2014.959216.

[23] Sheerin F, Eustace-Cook J, Wuytack F, Doyle C. Medication management in intellectual disability settings: A systematic review. J Intellect Disabil. 2021;25:242–76. 10.1177/1744629519886184. 31735106 10.1177/1744629519886184

[24] Smith MVA, Adams D, Carr C, Mengoni SE. Do people with intellectual disabilities understand their prescription medication? A scoping review. J Appl Res Intellect Disabil. 2019;32:1375–88. 10.1111/jar.12643. 31338972 10.1111/jar.12643 PMC6852265

[25] Ghosh I, Adams D, Auguste P, Brown A, Chaplin E, Flynn S, et al. Challenges of using and managing medication: a meta-ethnography of the experiences and perceptions of people with intellectual disability and people who support them. BMJ Open. 2025;15:e090876. 10.1136/bmjopen-2024-090876. 40973385 10.1136/bmjopen-2024-090876 PMC12458619

[26] Flood B, Henman MC. Experiences of the medication use process by people with intellectual disabilities. What Pharmacist Should Know! Pharm (Basel). 2021. 10.3390/pharmacy9010024. 10.3390/pharmacy9010024 PMC7838794 33494475

[27] Nabhanizadeh A, Oppewal A, Boot FH, Maes-Festen D. Effectiveness of medication reviews in identifying and reducing medication-related problems among people with intellectual disabilities: A systematic review. J Appl Res Intellect Disabil. 2019;32:750–61. 10.1111/jar.12580. 30793852 10.1111/jar.12580 PMC6850346

[28] Bergqvist M, Ulfvarson J, Karlsson EA. Nurse-led medication reviews and the quality of drug treatment of elderly hospitalized patients. Eur J Clin Pharmacol. 2009;65:1089–96. 10.1007/s00228-009-0728-2. 19798491 10.1007/s00228-009-0728-2

[29] Ferguson L, Murphy GH. The effects of training on the ability of adults with an intellectual disability to give informed consent to medication. J Intellect Disabil Res. 2014;58:864–73. 10.1111/jir.12101. 24341991 10.1111/jir.12101

[30] Kuntz JL, Safford MM, Singh JA, Phansalkar S, Slight SP, Her QL, et al. Patient-centered interventions to improve medication management and adherence: a qualitative review of research findings. Patient Educ Couns. 2014;97:310–26. 10.1016/j.pec.2014.08.021. 25264309 10.1016/j.pec.2014.08.021 PMC5830099

[31] Nadolny S, Bruland D, Grunwald M, Gröndahl A, Grammatico J, Richter MT, et al. Case management and care expertise as a prevention approach for adults with intellectual disabilities (FaPP-MgB): study protocol for a randomized-controlled trial. Trials. 2023;24:136. 10.1186/s13063-023-07155-w. 36814350 10.1186/s13063-023-07155-w PMC9946867

[32] Hawe P, Shiell A, Riley T. Complex interventions: how out of control can a randomised controlled trial be? BMJ. 2004;328:1561–3. 10.1136/bmj.328.7455.1561. 15217878 10.1136/bmj.328.7455.1561 PMC437159

[33] Vos R, Boesten J, van den Akker M. Fifteen-year trajectories of Multimorbidity and polypharmacy in Dutch primary care-A longitudinal analysis of age and sex patterns. PLoS ONE. 2022;17:e0264343. 10.1371/journal.pone.0264343. 35213615 10.1371/journal.pone.0264343 PMC8880753

[34] Lonchampt S, Gerber F, Aubry J-M, Desmeules J, Kosel M, Besson M. Prevalence of polypharmacy and inappropriate medication in adults with intellectual disabilities in a hospital setting in Switzerland. Front Psychiatry. 2021;12:614825. 10.3389/fpsyt.2021.614825. 34248693 10.3389/fpsyt.2021.614825 PMC8267250

[35] Doherty AJ, Atherton H, Boland P, Hastings R, Hives L, Hood K, et al. Barriers and facilitators to primary health care for people with intellectual disabilities and/or autism: an integrative review. BJGP Open. 2020. 10.3399/bjgpopen20X101030. 32605913 10.3399/bjgpopen20X101030 PMC7465578

[36] Marks B, Sisirak J, Heller T. Health promotion and people with intellectual disability. In: Prasher VP, Janicki MP, editors. Physical health of adults with intellectual and developmental disabilities. Cham: Springer International Publishing; 2019. pp. 359–79.

[37] Roll AE. Health promotion for people with intellectual disabilities - A concept analysis. Scand J Caring Sci. 2018;32:422–9. 10.1111/scs.12448. 28497855 10.1111/scs.12448

[38] Barre K, Boettcher AM, Schüßler N, Weber P. Qualifizierung für innovative aufgaben in der Pflege. PADUA. 2020;15:297–302. 10.1024/1861-6186/a000582.

[39] Eldridge S, Kerry SM. A practical guide to cluster randomised trials in health services research. Chichester, West Sussex: Wiley; 2012.

[40] Uschner D, Schindler D, Hilgers R-D, Heussen N. RandomizeR: an R package for the assessment and implementation of randomization in clinical trials. J Stat Soft. 2018. 10.18637/jss.v085.i08.

[41] Mahler C, Hermann K, Horne R, Ludt S, Haefeli WE, Szecsenyi J, Jank S. Assessing reported adherence to Pharmacological treatment recommendations. Translation and evaluation of the medication adherence report scale (MARS) in Germany. J Eval Clin Pract. 2010;16:574–9. 10.1111/j.1365-2753.2009.01169.x. 20210821 10.1111/j.1365-2753.2009.01169.x

[42] Horne R, Weinman J. Self-regulation and Self-management in asthma: exploring the role of illness perceptions and treatment beliefs in explaining Non-adherence to preventer medication. Psychol Health. 2002;17:17–32. 10.1080/08870440290001502.

[43] Greiner W, Weijnen T, Nieuwenhuizen M, Oppe S, Badia X, Busschbach J, et al. A single European currency for EQ-5D health states. Results from a six-country study. Eur J Health Econ. 2003;4:222–31. 10.1007/s10198-003-0182-5. 15609189 10.1007/s10198-003-0182-5

[44] Stange D, Kriston L, Langebrake C, Cameron LK, Wollacott JD, Baehr M, Dartsch DC. Development and psychometric evaluation of the German version of the medication regimen complexity index (MRCI-D). J Eval Clin Pract. 2012;18:515–22. 10.1111/j.1365-2753.2011.01636.x. 21320239 10.1111/j.1365-2753.2011.01636.x

[45] George J, Phun Y-T, Bailey MJ, Kong DCM, Stewart K. Development and validation of the medication regimen complexity index. Ann Pharmacother. 2004;38:1369–76. 10.1345/aph.1D479. 15266038 10.1345/aph.1D479

[46] Ustun TB, Kostanjesek N, Chatterji S, Rehm J. World Health Organization (WHO). Measuring health and disability: manual for WHO disability assessment schedule (WHODAS 2.0). Geneva: World Health Organization (WHO); 2010.

[47] Faul F, Erdfelder E, Buchner A, Lang A-G. Statistical power analyses using G*Power 3.1: tests for correlation and regression analyses. Behav Res Methods. 2009;41:1149–60. 10.3758/BRM.41.4.1149. 19897823 10.3758/BRM.41.4.1149

[48] Hughes CM. Compliance with medication in nursing homes for older people: resident enforcement or resident empowerment? Drugs Aging. 2008;25:445–54. 10.2165/00002512-200825060-00001. 18540686 10.2165/00002512-200825060-00001

[49] Parsons C, Lapane K, Kerse N, Hughes C. Prescribing for older people in nursing homes: a review of the key issues. Int J Older People Nurs. 2011;6:45–54. 10.1111/j.1748-3743.2010.00264.x. 21303465 10.1111/j.1748-3743.2010.00264.x

[50] Park J. Selbstbestimmtes Leben für Menschen mit geistiger Behinderung im betreuten Wohnen [Dissertation]. Marburg: Philipps-Universität Marburg; 2014.

[51] Thimm A, Dieckmann F, Haßler T. In which residential settings do older persons with intellectual disability live? A quantitative comparison of age groups for Westphalia-Lippe. Z Gerontol Geriatr. 2019;52:220–7. 10.1007/s00391-019-01533-3. 30911834 10.1007/s00391-019-01533-3

[52] Maetzel J, Heimer A, Braukmann J, Frankenbach P, Ludig L, Schmutz S. Dritter Teilhabebericht der bundesregierung über die lebenslagen von menschen Mit Beeinträchtigungen: Teilhabe - Beeinträchtigung - Behinderung. Bonn: Bundesministerium für Arbeit und Soziales, Referat Information, Monitoring, Bürgerservice, Bibliothek; 2021.

[53] Gould E, Mitty E. Medication adherence is a partnership, medication compliance is not. Geriatr Nurs. 2010;31:290–8. 10.1016/j.gerinurse.2010.05.004. 20682408 10.1016/j.gerinurse.2010.05.004

[54] Nguyen T-M-U, La Caze A, Cottrell N. What are validated self-report adherence scales really measuring? A systematic review. Br J Clin Pharmacol. 2014;77:427–45. 10.1111/bcp.12194. 23803249 10.1111/bcp.12194 PMC3952718

[55] Patel I, Erickson SR, Caldwell CH, Woolford SJ, Bagozzi RP, Chang J, Balkrishnan R. Predictors of medication adherence and persistence in medicaid enrollees with developmental disabilities and type 2 diabetes. Res Social Adm Pharm. 2016;12:592–603. 10.1016/j.sapharm.2015.09.008. 26522400 10.1016/j.sapharm.2015.09.008

[56] Cyrus AC, Royer J, Carroll DD, Courtney-Long EA, McDermott S, Turk MA. Anti-Hypertensive medication use and factors related to adherence among adults with intellectual and developmental disabilities. Am J Intellect Dev Disabil. 2019;124:248–62. 10.1352/1944-7558-124.3.248. 31026202 10.1352/1944-7558-124.3.248 PMC6554647

[57] Vacek JL, Hunt SL, Shireman T. Hypertension medication use and adherence among adults with developmental disability. Disabil Health J. 2013;6:297–302. 10.1016/j.dhjo.2013.02.003. 24060252 10.1016/j.dhjo.2013.02.003

[58] Zaal RJ, Ebbers S, Borms M, de Koning B, Mombarg E, Ooms P, et al. Medication review using a systematic tool to reduce inappropriate prescribing (STRIP) in adults with an intellectual disability: A pilot study. Res Dev Disabil. 2016;55:132–42. 10.1016/j.ridd.2016.03.014. 27065309 10.1016/j.ridd.2016.03.014

[59] Erickson SR, Nicaj D, Barron S. Complexity of medication regimens of people with intellectual and developmental disabilities. J Intellect Dev Disabil. 2018;43:351–61. 10.3109/13668250.2017.1350836.

[60] O’Dwyer M, Peklar J, McCallion P, McCarron M, Henman MC. Factors associated with polypharmacy and excessive polypharmacy in older people with intellectual disability differ from the general population: a cross-sectional observational nationwide study. BMJ Open. 2016;6:e010505. 10.1136/bmjopen-2015-010505. 27044582 10.1136/bmjopen-2015-010505 PMC4823458

[61] O’Connell J, Burke É, Mulryan N, O’Dwyer C, Donegan C, McCallion P, et al. Drug burden index to define the burden of medicines in older adults with intellectual disabilities: an observational cross-sectional study. Br J Clin Pharmacol. 2018;84:553–67. 10.1111/bcp.13479. 29193284 10.1111/bcp.13479 PMC5809360

[62] Doan TN, Lennox NG, Taylor-Gomez M, Ware RS. Medication use among Australian adults with intellectual disability in primary healthcare settings: a cross-sectional study. J Intellect Dev Disabil. 2013;38:177–81. 10.3109/13668250.2013.778968. 23550741 10.3109/13668250.2013.778968

[63] de Kuijper G, Hoekstra P, Visser F, Scholte FA, Penning C, Evenhuis H. Use of antipsychotic drugs in individuals with intellectual disability (ID) in the netherlands: prevalence and reasons for prescription. J Intellect Disabil Res. 2010;54:659–67. 10.1111/j.1365-2788.2010.01275.x. 20426795 10.1111/j.1365-2788.2010.01275.x

[64] Henman M, O’Dwyer M, Paruk M, Connell M. Complexity of medication use in people with intellectual disabilities.: IASSIDD world Congress, Melbourne. J Intellect Disabil Res. 2016;60:681. 10.1111/jir.12305.

[65] Saqr Y, Braun E, Porter K, Barnette D, Hanks C. Addressing medical needs of adolescents and adults with autism spectrum disorders in a primary care setting. Autism. 2018;22:51–61. 10.1177/1362361317709970. 28750547 10.1177/1362361317709970 PMC5788079

